# Rate of Homologous Desensitization and Internalization of the GLP-1 Receptor

**DOI:** 10.3390/molecules22010022

**Published:** 2016-12-26

**Authors:** Ghina Shaaban, Mabayoje Oriowo, Suleiman Al-Sabah

**Affiliations:** Department of Pharmacology & Toxicology, Faculty of Medicine, Kuwait University, PO Box 24923, 13110 Safat, Kuwait; ginaboss72@gmail.com (G.S.); oriowo@hsc.edu.kw (M.O.)

**Keywords:** GLP-1, receptor, desensitization, internalization

## Abstract

The glucagon-like peptide-1 receptor (GLP-1R) is an important target in the treatment of type 2 diabetes mellitus. The aim of this study was to compare the rate of agonist stimulated desensitization and internalization of GLP-1R. To this end, an N-terminally myc-tagged GLP-1R was stably expressed in HEK-293 cells. Homologous desensitization was assessed by measuring the cAMP response to agonist stimulation following pre-incubation with agonist for up to 120 min. Receptor internalization was monitored using an indirect ELISA-based method and confocal microscopy. Pre-incubation with GLP-1 resulted in a time-dependent loss of response to a second stimulation. Washing cells following pre-incubation failed to bring cAMP levels back to basal. Taking this into account, two desensitization rates were calculated: “apparent” (t_1/2_ = 19.27 min) and “net” (t_1/2_ = 2.99 min). Incubation of cells with GLP-1 also resulted in a time-dependent loss of receptor cell surface expression (t_1/2_ = 2.05 min). Rapid agonist-stimulated internalization of GLP-1R was confirmed using confocal microscopy. Stimulation of GLP-1R with GLP-1 results in rapid desensitization and internalization of the receptor. Interestingly, the rate of “net” desensitization closely matches the rate of internalization. Our results suggest that agonist-bound GLP-1R continues to generate cAMP after it has been internalized.

## 1. Introduction

Glucagon-like peptide-1 (GLP-1) is a peptide hormone secreted from intestinal endocrine L-cells in response to nutrient ingestion. GLP-1’s most well-characterized biological function is to potentiate glucose-dependent insulin secretion, although it has other effects, such as suppressing appetite and glucagon secretion, which make the GLP-1 receptor (GLP-1R) an attractive target in the treatment of type 2 diabetes mellitus [1,2,3,4,5]. However, a characteristic of type 2 diabetes is an impairment in GLP-1 sensitivity, although exogenous administration of long-acting GLP-1 analogues can overcome this impairment [6]. Indeed, several anti-diabetic drugs in clinical use are GLP-1R agonists [7,8]. GLP-1R is a member of the secretin family of G protein-coupled receptors (GPCRs), and couples positively to Gs to activate adenylate cyclase and increase intracellular cAMP [9]; it is also capable of coupling to other G proteins, such as Gq [10].

Chronic exposure of a receptor to agonist can result in the loss of response to subsequent stimulation—a process termed homologous desensitization [11]. For some GPCRs—such as the β2 adrenoceptor (β2AR)—homologous desensitization is a two-step process. First, the agonist-occupied receptor is phosphorylated at specific serine and threonine residues in the receptor’s C-terminal tail and 3rd intracellular loop region by GPCR kinases (GRKs). This allows a family of proteins known as arrestins to bind to the receptor, preventing it from interacting with G proteins, thus desensitizing the G protein-dependent component [12,13]. Arrestin also facilitates internalization of the receptor by interacting with clathrin [14]. The mechanisms by which GLP-1R undergoes desensitization and internalization are less well studied. Although activation of GLP-1R results in arrestin recruitment to the receptor [15,16], a recent study has demonstrated that agonist-induced internalization of GLP-1R is dependent on caveolin-1 and dynamin, and independent of arrestin [17]. In this study, we aimed to compare the rate of GLP-1-mediated receptor desensitization and internalization in order to further understand the signaling properties of this clinically important receptor.

## 2. Results

### 2.1. Validation of N-Terminally Labelled GLP-1R

**Figure 1:**
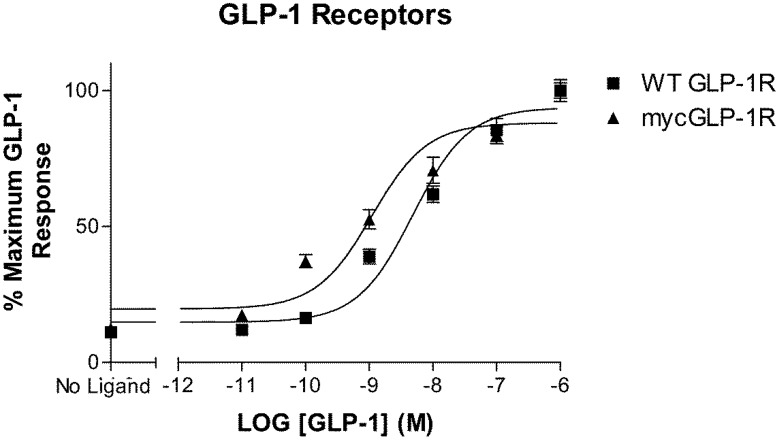
Concentration-response curves for glucagon-like peptide-1 (GLP-1) at wild-type (WT) and myc-tagged GLP-1 receptor (mycGLP-1R) transiently expressed in HEK-293 cells. The curves represent one of at least three independent experiments, and each point represents the mean of triplicates, with the S.E.M. displayed as error bars. Counts were normalized to the maximum GLP-1 response.

Introduction of a myc-tag to the N-terminus of GLP-1R immediately downstream of the putative signal peptide did not significantly affect the potency of GLP-1 at this receptor when assessed using a cAMP-responsive luciferase assay (Figure 1). GLP-1 stimulated wild-type (WT) and myc-tagged GLP-1R with a *p*EC_50_ value of 8.3 ± 0.1 (n = 3) and 8.8 ± 0.1 (n = 3), respectively.

### 2.2. Homologous Desensitization of GLP-1R

**Figure 2:**
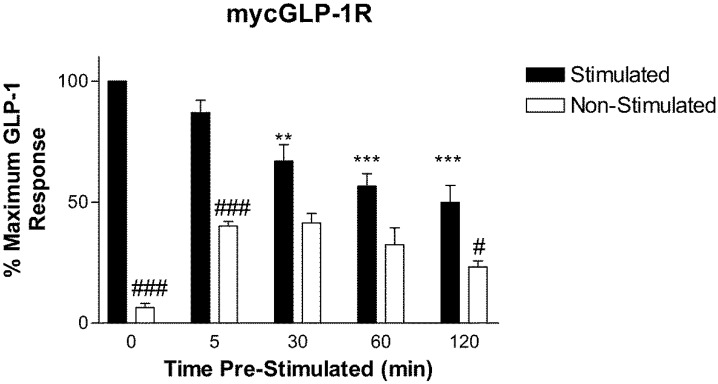
Desensitization of mycGLP-1R to a second stimulation with 1 µM GLP-1 following increasing periods of pre-stimulation with 1 µM GLP-1. Data are expressed as a percentage of maximum GLP-1 response and represent the mean, with the S.E.M. displayed as error bars from three independent experiments. ** *p* < 0.005, *** *p* < 0.001: GLP-1 response significantly different from control (no pre-stimulation). ^#^ *p* < 0.05, ^###^ *p* < 0.001: Non-Stimulated significantly different the Stimulated following the same period of pre-stimulation.

**Figure 3:**
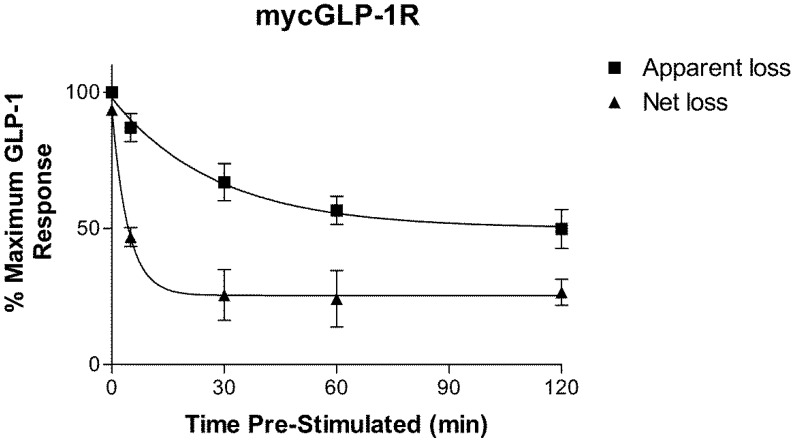
Rate of desensitization of mycGLP-1R. Two rates of desensitization were calculated. Apparent loss represents the loss of response to 1 µM GLP-1 following pre-incubation with 1 µM GLP-1. Net loss represents the loss of response to GLP-1 minus the non-stimulated control following pre-incubation with GLP-1. Data are expressed as a percentage of maximum GLP-1 response and represent the mean, with the S.E.M. displayed as error bars from three independent experiments.

Pre-incubation of HEK-293 cells stably expressing mycGLP-1R with 1 µM GLP-1 for 30 min or longer significantly reduced the cAMP response to a second stimulation with 1 µM GLP-1. The extent of desensitization was time dependent (Figure 2). As washing following the first stimulation did not bring cAMP levels back to basal levels, two rates of desensitization were calculated. The first was calculated using the response to a second stimulation with GLP-1 following increasing periods of pre-incubation with GLP-1 (termed “apparent loss”). Using this method, GLP-1R desensitized with a rate of k = 0.036 ± 0.01 s^−1^ (n = 3) (t_1/2_ = 19.27 min). The second rate was calculated using the response to a second stimulation with GLP-1 minus the non-stimulated value (termed “net loss”); that is, net loss of response to a second stimulation (Figure 3). Using the second method, GLP-1R desensitized with a rate of k = 0.23 ± 0.06 s^−1^ (n = 3) (t_1/2_ = 2.99 min). The second (net loss) rate was significantly (*p* < 0.05) faster than the first (apparent loss).

### 2.3. Internalization of GLP-1R

**Figure 4:**
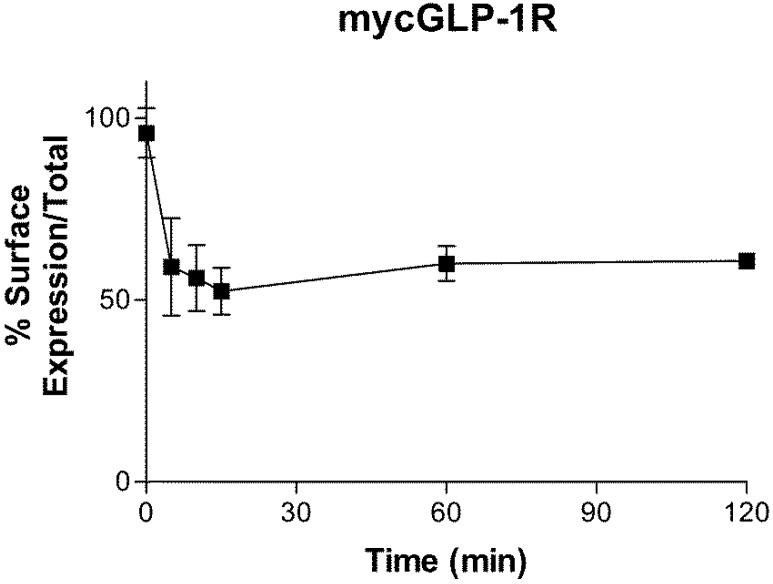
Agonist-induced internalization of mycGLP-1R. Incubation with 1 µM GLP-1 caused a time-dependent loss of cell surface expression of mycGLP-1R. Data are expressed as a percentage of receptor surface expression over total expression at time zero and represent the mean, with the S.E.M. displayed as error bars from three independent experiments.

Incubation of HEK-293 cells stably expressing mycGLP-1R with 1 µM GLP-1 caused a rapid loss of receptor cell surface expression (Figure 4). The first 15 min were used to calculate the initial rate of receptor internalization, as a small reversal was seen at the next time point (although this did not reach significance). mycGLP-1R internalized with an initial rate of k = 0.33 ± 0.1 s^−1^ (n = 3) (t_1/2_ = 2.05 min).

### 2.4. Visualization of Internalized GLP-1R

**Figure 5:**
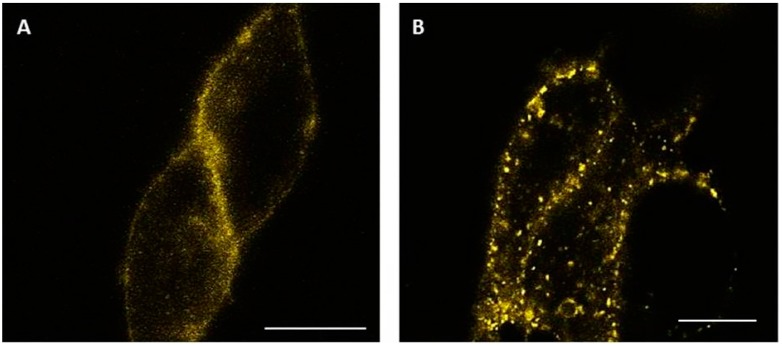
Agonist induced internalization of GLP-1R-YFP. Confocal images were taken before (**A**) and 5 min after (**B**) stimulating cells expressing GLP-1R-YFP with 1 µM GLP-1. Scale bar = 10 µm.

HEK-293 cells transiently transfected with GLP-1R-Yellow Fluorescent Protein (YFP) were plated on poly-d-lysine-coated coverslips and observed using a confocal microscope. Prior to agonist-stimulation, the receptor was located at the plasma membrane (Figure 5A). Five minutes after stimulating cells with 1 µM GLP-1, the receptor appeared as punctate dots in the cytoplasm (Figure 5B). Images are representative of at least three experiments.

## 3. Discussion

In the present study, we compare the rate of agonist-induced desensitization and internalization of GLP-1R. To monitor cell surface expression of the receptor, we used a modified receptor with a myc-tag inserted at the N-terminus, down-stream of the receptor’s signal peptide, as it is cleaved during post-translation processing and trafficking [18]. Using a cAMP-responsive luciferase assay, we show that this modification did not significantly alter the potency of GLP-1 at the receptor, which is in agreement with earlier work [15]. Pre-exposure of cells to 1 µM GLP-1 resulted in a time-dependent loss of response to subsequent stimulation. GLP-1R has previously been shown to undergo homologous desensitization [19,20,21], and although this process has been described as rapid, we are unaware of a study that gives its rate. We observed that washing the cells after pre-incubation with agonist did not bring cAMP levels back to basal. This could be because the washing step was insufficient to remove all the ligand from the receptor or because the receptor had undergone endocytosis still bound to the ligand, where it continued to signal. As a result of this observation, we calculated two rates of desensitization. The first, slower, rate represents the apparent loss of response. The second, faster, rate accounts for the levels of cAMP in the non-stimulated cells following pre-stimulation, and represents net loss of response. 

Incubation of cells stably expressing mycGLP-1R with 1 µM GLP-1 also results in a time-dependent loss of cell surface expression of the receptor. This was confirmed using a YFP-labelled receptor in confocal microscopy experiments, where internalization of the receptor was observed after 5 min of exposure to agonist. The rate we calculated for receptor internalization is in the same range (t_1/2_ = 2–3 min) as previous studies using radioligand binding [22] and time-resolved fluorescence resonance energy transfer [23]. Interestingly this rate is almost ten times faster than the “apparent” rate of desensitization, but similar to the rate of “net” desensitization. 

Homologous receptor desensitization and internalization are two distinct processes. Removal of the GRK2 phosphorylation sites of the β2AR impairs desensitization, but not internalization [24]. Conversely, a mutated β2AR that is unable to internalize can still be desensitized [25]. Phosphorylation of β2AR by GRKs is a prerequisite for arrestin binding [26]. Arrestin binding prevents the receptor from interacting with G proteins, but also allows β2AR to interact with the cell’s internalization apparatus. Although GLP-1R has been shown to interact with arrestin [15,16], both desensitization and internalization appear to be arrestin-independent processes [27,28]. Using fluorescently-labelled ligands and receptors, Kuna and colleagues were able to demonstrate that agonist-bound GLP-1R co-localized with adenylate cyclase in endosomes. Furthermore, inhibition of GLP-1R internalization was shown to attenuate cAMP production in BRIN-BD11 cells [29]. Generation of cAMP following endocytosis has also been shown for the glucagon receptor [30], parathyroid hormone receptor (PTHR) [31], and importantly the second incretin hormone receptor—the glucose-dependent insulinotropic polypeptide receptor (GIPR) [32]. Like GLP-1R, these receptors belong to the secretin family of GPCRs. For PTHR, this sustained production of cAMP following internalization has been reported to be mediated by arrestin2 [33]. While this has not been demonstrated for GLP-1R, knockdown of arrestin2 in cultured INS-1 pancreatic β-cells results in a reduction in cAMP levels and insulin secretion [27]. Intriguingly, this may not be the case for GIPR, as this receptor has been shown to be unable to recruit arrestins [15,34].

Our desensitization data can be explained in light of these recent findings. The persistent cAMP production we observe following washing can be explained by an internalized agonist-bound receptor continuing to signal. Future experiments using endocytosis inhibitors will hopefully support this hypothesis. When we take this signal into account and subtract it from the signal generated by the second stimulation, the “net” rate of desensitization matches the rate of internalization. This suggests that the “net” loss of response to a second stimulation is due to a reduction in the number of receptors at the cell surface, and that the slower “apparent” rate of desensitization is due to the contribution of cAMP generated following receptor internalization. As GLP-1R is known to recruit arrestin, it is tempting to speculate that—like for PTHR—it may play a role in this phenomenon.

The present study is limited by the use of a single dose of GLP-1 to trigger receptor desensitization and internalization. A recent study that utilized a time-resolved Förster Resonance Energy Transfer (FRET)-based assay to monitor GLP-1R internalization in real-time demonstrated that the kinetics of ligand-induced receptor internalization were dose dependent [23]. It would be interesting to investigate the effect of dose on the rate of receptor desensitization, as this would provide a useful correlation between receptor desensitization and internalization. The mechanisms by which GLP-1R is homologously desensitized and internalized remain to be fully elucidated, although a recent study has demonstrated that agonist-stimulated internalization of GLP-1R is mediated by the Gαq pathway [17]. Further studies are warranted in order to explore this area of receptor signaling, extend it to pancreatic β-cells and possibly animal models of type 2 diabetes, and identify the molecular mechanisms that drive it—especially as a diminished response to GLP-1 is a characteristic of type 2 diabetes mellitus.

## 4. Materials and Methods

### 4.1. Construction of cDNA

cDNA encoding the wild type (WT), myc-tagged, and YFP-tagged human GLP-1R have been previously described [15]. The myc-tag was introduced to the N-terminus of GLP-1R directly downstream of the putative signal peptide to produce mycGLP-1R. GLP-1R was C-terminally labelled with YFP to give GLP-1R-YFP.

### 4.2. Cell Culture and Transfection of Cells

Human embryonic kidney 293 (HEK-293) cells (ECACC Cat. No. 85120602) were cultured in Dulbecco’s modified Eagle’s media supplemented with 10% fetal calf serum, 100 U/mL penicillin, and 100 µg/mL streptomycin. The cells were maintained at 37 °C in a humidified environment containing 5% CO_2_. HEK-293 cells were transiently transfected using Effectene (Qiagen, Hilden, Germany), according to the manufacturer’s instructions. A stable cell line expressing mycGLP-1R was generated also using Effectene and selecting transfected clones by the addition of 800 μg·mL^−1^ G418 antibiotic.

### 4.3. Luciferase Assay

WT and myc-tagged GLP-1R activation was assessed using a luciferase reporter gene assay, as described previously [35]. Briefly, HEK-293 cells were transiently transfected with cDNA encoding GLP-1R and a reporter gene construct consisting of a cAMP-response element fused to a reporter gene encoding firefly luciferase (Cre-luc) at a ratio of 1:2. Twenty-four hours after transfection, the cells were seeded into white 96-well plates (Thermo Scientific, Roskilde, Denmark) at a density of 10,000 cells/well. Twenty-four hours later, the cells were incubated for 3 h in medium containing GLP-1 and then lysed. Luciferase activity was quantified using LucLite reagent (PerkinElmer Life and Analytic Sciences, Wellesley, MA, USA).

### 4.4. Desensitization Assay

HEK-293 cell stably expressing mycGLP-1R were seeded into poly-d-lysine-coated 96-well plates at a density of 10,000 cells/well. Twenty-four hours later, the cells were pre-stimulated with 1 µM GLP-1 for 0, 5, 30, 60, and 120 min at 37 °C. Cells were then washed three times with media and stimulated with 1 µM GLP-1 in media containing 500 µM IBMX for 15 min. Cells were then lysed and cAMP was measured by ELISA (cAMP Biotrak Enzyme ImmunoAssay Kit, GE Healthcare, Little Chalfont, UK). The scheme of this assay is shown in Table 1. The rate of “apparent loss” of signal was calculated using the “stimulated” bars shown in Figure 2. The rate of “net loss” of signal was calculated by subtracting the “non-stimulated” control from “stimulated”.

### 4.5. Internalization Assay

Cell surface expression of mycGLP-1R was determined using an indirect ELISA-based assay. HEK-293 cells stably expressing mycGLP-1R were seeded into poly-d-lysine-coated 96-well plates at a density of 10,000 cells/well. Twenty-four hours later, the cells were stimulated with 1 µM GLP-1 for 0, 5, 10, 15, 60, and 120 min at 37 °C. Cells were then washed three times with PBS and fixed with 4% ice-cold paraformaldehyde for 10 min. After washing again, “Total” receptor expression was determined by permeabilizing the cells with 0.1% triton-100X for 2 min and washing with 100 mM glycine in PBS. “Surface” receptor expression was determined in the absence of detergent. Cells were then incubated in blocking solution (2% bovine serum albumin in phosphate-buffered saline) for 30 min. A horseradish peroxidase-conjugated antibody directed against the myc-tag was then added to the cells (1:500) for 1 h. Cells were then washed 5 times with phosphate-buffered saline (PBS) and 50 µL peroxidase substrate solution (3,3′,5-TetraMethylBenzidine, TMB) was added to each well and incubated for 30 min. The reaction was terminated by the addition of 50 µL 2 M H_2_SO_4_, and absorbance was measured at 450 nm.

### 4.6. Confocal Microscopy

HEK-293 cells transiently expressing GLP-1R-YFP were plated on to a poly-d-lysin-coated coverslip and mounted on to an “Attofluor” holder (Molecular Probes, Leiden, The Netherlands). The cellular location of GLP-1R-YFP was monitored by live cell confocal microscopy performed on a Zeiss LSM 510 meta system (Carl Zeiss, Oberkochen, Germany). YFP was excited with the 514 nm line of an argon laser, and images were taken with an oil-immersion 63× lens using the factory settings for YFP.

### 4.7. Data Analysis

The dose–response data were fitted to a sigmoidal curve using nonlinear regression, and the *p*EC_50_ (−logEC_50_) values calculated with the aid of GraphPad 3.0 (GraphPad, San Diego, CA, USA). Kinetics were calculated using the monoexponential function built into GraphPad 3.0. Statistical significance was calculated using a Student’s *t*-test or analysis of variance followed by a post hoc test (Bonferroni) where appropriate.

## 5. Conclusions

Our results show that stimulation of GLP-1R with GLP-1 results in rapid desensitization and internalization of the receptor. Interestingly, the rate of “net” desensitization closely matches the rate of internalization. Our results also suggest that agonist-bound GLP-1R continues to generate cAMP after it has been internalized. Future experiments should address this in greater molecular detail.

## Figures and Tables

**Table 1:** Schematic representation of the desensitization assay.

**Control (Media)** **↓**	**Control (Media)** **↓**	**Pre-Stimulate with 1 µM GLP-1 (5–120) min** **↓**	**Pre-Stimulate with 1 µM GLP-1 (5–120) min** **↓**
**Wash** **↓**	**Wash** **↓**	**Wash** **↓**	**Wash** **↓**
**Stimulate 1 µM GLP-1 15 min** **(Stimulated)** **↓**	**Media** **(Non-Stimulated)** **↓**	**Stimulate 1 µM GLP-1 15 min** **(Stimulated)** **↓**	**Media** **(Non-Stimulated)** **↓**
**Assay**	**Assay**	**Assay**	**Assay**
